# The associations between caregivers’ psychosocial characteristics and caregivers’ depressive symptoms in stroke settings: a cohort study

**DOI:** 10.1186/s40359-022-00828-2

**Published:** 2022-05-09

**Authors:** Yen Sin Koh, Mythily Subramaniam, David Bruce Matchar, Song-Iee Hong, Gerald Choon-Huat Koh

**Affiliations:** 1 Research Division, Institute of Mental Health, Singapore, Singapore; 2 Saw Swee Hock School of Public Health, National University of Singapore, Singapore, Singapore; 3 Health Services and Systems Research, Duke-NUS Medical School, Singapore, Singapore; 4 Department of Social Welfare, Dongguk University, Seoul, South Korea; 5 Department of Medicine (General Internal Medicine) and Pathology, Duke University, Durham, North Carolina USA

**Keywords:** Caregiver, Depressive symptoms, Stroke, Survivor, Psychosocial, Patient-caregiver dyads

## Abstract

**Background:**

Studies have found that caregivers can influence stroke survivors’ outcomes, such as mortality. It is thus pertinent to identify significant factors associated with caregivers’ outcomes. The study objective was to examine the associations between caregivers’ psychosocial characteristics and caregivers’ depressive symptoms.

**Methods:**

The analysis obtained three-month and one-year post-stroke data from the Singapore Stroke Study, which was collected from hospital settings. Caregivers’ depressive symptoms were assessed via the Center for Epidemiologic Studies Depression instrument. Psychosocial characteristics of caregivers included subjective burden (Zarit Burden Interview), quality of care-relationship (a modified 3-item scale from the University of Southern California Longitudinal Study of Three-Generation Families) and expressive social support (an 8-item scale from Pearlin et al.). Mixed effect Tobit regressions were used to examine the associations between these study variables.

**Results:**

A total of 214 caregivers of stroke patients hospitalized were included in the final analysis. Most caregivers were Chinese women with secondary school education, unemployed and married to the patients. Caregivers' subjective burden was positively associated with their depressive symptoms (Partial regression coefficient: 0.18, 95% CI 0.11–0.24). Quality of care-relationship (Partial regression coefficient: − 0.35, 95% CI − 0.63 to − 0.06) and expressive social support (partial regression coefficient: − 0.28, 95% CI − 0.37 to − 0.19) were negatively associated with caregivers’ depressive symptoms. Caregivers’ depressive symptoms were higher at three-month post-stroke than one-year post-stroke (Partial regression coefficient: − 1.00, 95% CI − 1.80 to − 0.20).

**Conclusion:**

The study identified subjective burden, quality of care-relationship and expressive social support as significantly associated with caregivers’ depressive symptoms. Caregivers’ communication skills may also play a role in reducing caregivers’ depressive symptoms.

**Supplementary Information:**

The online version contains supplementary material available at 10.1186/s40359-022-00828-2.

## Introduction

About 50% of stroke survivors live with physical and mental disabilities worldwide [1]. With up to 80% of patients going back to the community after acute hospitalisation, a caregiver is often needed to assist them [1]. Caregivers may face sudden changes to their lives as stroke can occur unpredictably. For instance, they may need to reduce their working hours and social activities to care for stroke survivors [2]. Moreover, the additional physical demands of caregiving may lead to fatigue and sleep disruption [2]. These caregiving-related challenges can affect caregivers physically and psychologically.

In particular, caregivers may experience depressive symptoms from their additional responsibilities. A systematic review by Loh et al. [3], which included 11 studies, unveiled that the global prevalence of depressive symptoms among caregivers of stroke survivors was 40.2%. Other studies also showed that this prevalence ranged between 27 and 59.5% [4–6]. The relatively high prevalence is concerning, as caregivers with depressive symptoms can be detrimental to patient-related outcomes. For instance, depression of caregivers was associated with increased odds of 6-month mortality of stroke survivors [7]. Caregivers with depressive symptoms were also more likely to increase the risk of patients' depressive symptoms [8] and institutionalization [9].

Models have been developed to understand how several variables, such as sociodemographic, may influence caregivers’ depressive symptoms [10]. For instance, in Pearlin’s Stress Process Model [11], the outcomes of stress (e.g., depression) depend on the context (e.g., sociodemographic of the patient-caregiver dyads), primary and secondary stressors, and psychosocial resources (e.g., coping and social support). Primary stressors are those that occur from the patient-caregiver relationship, whereas secondary stressors are those that arise from outside of the caregiving situation. This model has guided several studies to understand the associations between variables of interest and caregivers’ depressive symptoms [10, 12].

However, existing stroke-related studies have focused on the associations between caregivers’ depressive symptoms with the demographics and physical characteristics of patient-caregiver dyads [3, 13]. Studies examining the associations between caregivers’ depressive symptoms and caregivers’ psychosocial characteristics (e.g., subjective burden, expressive social support and quality of care-relationship between patient and caregiver) are limited in stroke settings [13]. For instance, a systematic review by del-Pino-Casado et al. [14] that examined the association between subjective burden and caregivers’ depressive symptoms identified 32 dementia-related studies but only five stroke-related studies.

Related studies, particularly from other settings, have shown significant associations between caregivers’ depressive symptoms and the caregivers’ psychosocial characteristics. In the same systematic review by del-Pino-Casado et al. [14], the association between subjective burden and caregivers’ depressive symptoms was positive, especially in dementia and stroke settings. Litwin et al. [15] also found that having a good patient-caregiver relationship can reduce caregivers’ depressive symptoms among older caregivers. Since caregivers' psychosocial characteristics can change, identifying significant psychosocial characteristics in stroke settings can help healthcare providers develop interventions that alleviate caregivers’ depressive symptoms [13]. Furthermore, since stroke survivors have a higher risk of developing dementia [1], effective interventions can prepare caregivers for this situation.

Singapore is a country in South-East Asia with a multi-ethnic population of 75.9% Chinese, 15.0% Malay, 7.5% Indian and 1.6% other ethnicities [16]. Its ageing population [17] implies a greater incidence of chronic diseases such as stroke. The crude incidence rate of stroke had risen from 187.9 to 244.7 per 100,000 population between 2009 to 2018 [18]. Moreover, the likelihood of patients with stroke surviving has improved over the years. From 2011 to 2017. The age-adjusted death rate fell from 20.8 to 14.1 per 100,000 patients [19]. With the recent emphasis on community care [20], there is an increasing need for caregivers to look after stroke survivors in the community. More patient-caregiver dyads imply that managing caregiver outcomes is critical to improving stroke survivors’ outcomes in Singapore.

Our study used a longitudinal dataset in Singapore to examine (1) the prevalence of caregivers’ depressive symptoms at three-month and one-year post-stroke and (2) the associations between caregivers’ depressive symptoms and caregivers’ psychosocial characteristics (subjective burden, expressive social support and quality of care-relationship between patient and caregiver). Based on the literature, we hypothesised that these psychosocial characteristics are associated with caregivers’ depressive symptoms.

## Methods

Our study utilised a dataset acquired from a prospective cohort study, the Singapore Stroke Study (S3) [21]. Participants were recruited from December 2010 to September 2013 at five public hospitals: Changi General Hospital, Khoo Teck Puat Hospital, Tan Tock Seng Hospital, Singapore General Hospital and National University Hospital [21]. The inclusion criteria for the participants were (1) aged 40 years old and above, (2) Singaporeans or permanent residents, (3) residing in Singapore for the next one year, (4) diagnosed with stroke recently by a clinician and/or verified by CT/MRI brain scan and (5) not having global aphasia [21]. All patients who were eligible were approached for the study.

**Fig. 1:**
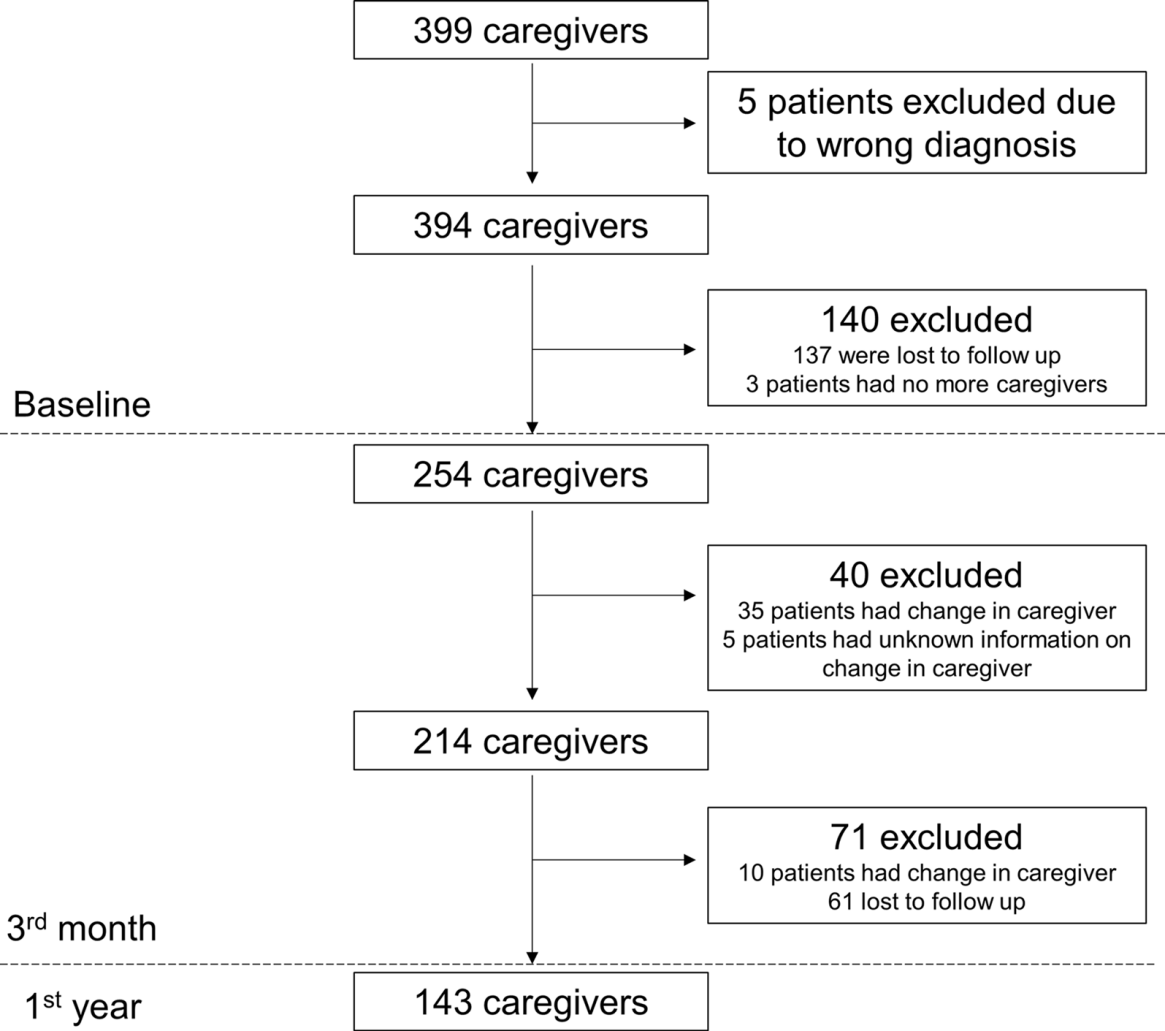
Study flow diagram

Our analysis only included participants who were diagnosed with stroke and had a caregiver. A caregiver could be a friend or family member, aged 21 years and older who tended to the patient's needs without financial compensation [22]. We excluded participants who changed caregiver, had no caregiver, or had no information on caregiver status at three-month post-stroke. If the patient changed caregiver at one-year post-stroke, our analysis excluded the caregiver's data at that time point. A total of 399 caregivers were identified from S3 (Fig. 1). After excluding caregivers who were ineligible, 214 caregivers were included in the analysis. At one-year post-stroke, ten patients changed caregivers, and 61 caregivers were lost-to-follow-up.

The S3 collected data from the patient and caregiver at five time points: baseline, three-month, six-month, nine-month and one-year post-stroke [21]. Our analysis focused on three-month and one-year post-stroke, as the variables of interest were available only at these time points. The data was collected via face-to-face interviews at three-month and one-year post-stroke [21].

Written informed consent was obtained from the patient and caregiver after explaining the study procedure [21]. The study was approved by the SingHealth Centralized Institutional Review Board (2010/724/A) and the National Health Group Domain Specific Review Board (A/10/690) [21].

### Outcome variable

The outcome variable was caregivers’ depressive symptoms, measured using the 11-item Center for Epidemiologic Studies Depression (CES-D) instrument [23] at three-month and one-year post-stroke. It allows respondents to rate various symptoms related to depression using a 3-point Likert scale, with 1 = None/Rarely to 3 = Often. The total score varies between 11 to 33, with a higher score indicating more depressive symptoms. A local study had previously utilised this instrument to measure caregivers’ depressive symptoms [23].

### Variables of interest

Our analysis examined the following caregivers’ psychosocial characteristics: subjective burden, expressive social support and quality of care-relationship between patient and caregiver. Subjective burden refers to the caregivers' adverse psychological and emotional reactions to their caregiving role [24]. Zarit Burden Interview (ZBI) was used to measure this construct. It is a 12-item instrument that allows respondents to rate negatively phrased questions on caregiving from 0 = Never to 4 = Nearly always [25]. The total score varies between 0 to 48, with a higher score indicating a higher subjective burden.

Expressive social support allows caregivers to share their experiences and express their emotions to others [26]. In the process, they can “share sentiments, seek understanding, vent frustration and build up self-esteem” [11]. It was measured using an 8-item scale devised by Pearlin et al. [11]. The responses were captured using a 4-point Likert Scale, with 1 = Strongly Disagree to 4 = Strongly Agree. The composite score ranges from 8 to 32, with a higher score representing better expressive social support. The variable had high internal consistency.

The quality of care-relationship between the patient and caregiver was measured using an instrument devised from the University of Southern California Longitudinal Study of Three-Generation Families [27]. It is a 4-item scale that measure “general closeness, communication, the similarity of views about life and degree of getting along” [27]. Responses were recorded with a 5-point Likert scale, with 1 = Not at all to 4 = Very. The total score ranges between 3 to 12, with a higher score representing a better patient-caregiver relationship. The third question (the similarity of views about life) was removed as local participants had difficulty comprehending it during pilot testing.

### Control variables

The established confounders included for the analysis were: (1) caregiver’s sex, (2) patient’s sex, (3) caregivers’ ethnicity, (4) caregiver's relationship with the patient, (5) patient’s age, (6) caregivers’ chronic conditions, (7) stroke survivor’s depressive symptoms and (8) objective burden. Variable (1)–(4) were significantly associated with caregiver's depressive symptoms in the systematic review by Loh et al. [3]. Variable (5)–(7) were significantly related to caregivers' depressive symptoms in a local study [8]. Several studies related to stroke and other conditions had suggested that caregivers' depressive symptoms were associated with their objective burden [28–30]. A stroke-related systematic review [14] also considered controlling for objective burden as a criterion for a well-designed study. Hence, our study included objective burden as a confounder.

For caregivers’ chronic conditions, caregivers were asked to self-report the presence of 21 health conditions. Following the methods from the local study [8], eight chronic conditions were selected: arthritis or rheumatism, asthma, cancer or leukemia, cataract, diabetes, heart problems, high blood pressure and kidney diseases. Stroke was not included in the analysis, as it was not captured in S3. The responses were classified into having no health conditions and having one or more health conditions. For stroke survivor’s depressive symptoms, it was measured using 11-item CES-D, which was similar to depressive symptoms of caregiver.

Objective burden includes the time and difficulty faced by caregivers in performing caregiving tasks [14, 31]. It was assessed using Oberst Caregiving Burden Score (OCBS), a 15-item instrument that allows caregivers to rate the time and difficulty for specific caregiving tasks [32]. In S3, the instrument measured only the time aspect. The responses were captured using a 5-point Likert scale, with 1 = none to 5 = A great amount. The total score ranged from 15 to 75, with a higher score indicating that caregivers spend a longer time doing the tasks.

### Statistical analysis

Patients’ and caregivers’ characteristics were presented at baseline. The following variables were included as time-varying variables: caregivers’ depressive symptoms, all variables of interest (subjective caregivers’ burden, expressive social support, quality of care-relationship), caregivers’ chronic conditions, stroke survivors’ depressive symptoms and caregivers’ objective burden. The summary statistics of these time-varying variables were presented at three-month and one-year post-stroke. Continuous variables were presented in either mean (standard deviation (SD)) or median (interquartile range (IQR)), depending on data distribution. Categorical variables were reported in frequencies and percentages.

Caregivers’ CES-D score was a censored variable, as the majority of the respondents had a score of 2 and below (three-month post-stroke: 33.0%, one-month post-stroke: 40.4%, Additional file 1: Fig. 1a, b). Therefore, our analysis used a mixed-effect Tobit regression model with a random intercept, which is suitable for censored outcome variables [33]. A random intercept was included to account for the possible intra-individual correlation in caregivers’ depressive symptoms at three-month and one-year post-stroke. This inclusion helped to estimate the individual intercepts for each caregiver. The mixed-effect regression model is adaptable to incomplete data in repeated measures [34], which means that caregivers with three-month post-stroke data but missing one-year post-stroke data (n = 71) can be included in the final model (n = 214).

All independent variables (baseline demographics, variables of interest and established control variables) were subjected to bivariate analysis to identify significant variables for the multivariate model. We then removed non-significant variables in the multivariable model using a stepwise approach with the Wald test. The final multivariable model consisted of all significant variables, controlling for the effect of time and established control variables. Partial regression coefficients and 95% confidence interval (CI) were presented for the multivariable model.

All analyses were performed using Stata/IC 16.0 (College Station, Texas), with a two-sided test at a significance level of 5%. The mixed-effect Tobit regression model was implemented using the *metobit* command [35]. Except for missing one-year data attributed to a change in caregiver or loss to follow-up, missing data were handled via complete case analysis.

## Results

At baseline (Table 1), the majority of the caregivers were female (72.4%), ethnic Chinese (53.8%), had secondary educational qualifications (41.8%), employed full-time (42.9%) and were the spouse to the patient (55.9%). The mean (SD) age of caregivers was 49.0 (13.0). The median (IQR) caregivers’ ZBI score was 11 (6–19), while the median (IQR) caregivers’ CES-D score was 7 (3–9).

**Table 1:** Summary statistics of patients’ and caregivers’ demographics at baseline (*n* = 214)

	*n* (%) (unless otherwise stated)
**Caregivers**^**a**^	
Mean age in years (SD)	49.0 (13.0)
Sex	
Male	59 (27.6%)
Female	155 (72.4%)
Ethnicity	
Chinese	114 (53.8%)
Malay	74 (34.9%)
Indian	19 (9.0%)
Others	5 (2.4%)
Educational qualification	
No qualification	8 (3.7%)
Primary	50 (23.4%)
Secondary	89 (41.8%)
Post-secondary/polytechnic	52 (24.4%)
University	14 (6.6%)
Employment status	
Employed full time	91 (42.9%)
Employed part time	22 (10.4%)
Unemployed	99 (46.7%)
Relationship with patient	
Spousal	119 (55.9%)
Children	74 (34.7%)
Others	20 (9.4%)
Median ZBI score (IQR)	11 (6—19)
Median CES-D (IQR)	7 (3—9)
**Patients**	
Mean age in year (SD)	62.8 (11.5)
Sex	
Male	137 (64.0%)
Female	77 (36.0%)
Ethnicity	
Chinese	119 (55.6%)
Malay	70 (32.7%)
Indian	21 (9.8%)
Others	4 (1.9%)

IQR, interquartile range; *n*, sample size; SD, standard deviation

^a^Number of missing observations: age (n = 13), ethnicity (n = 2), educational qualification (n = 1), employment status (n = 2), relationship with patient (n = 1), ZBI (n = 16), CES-D (n = 12)

Table 2 presents the summary statistics of the time-varying variables. The median caregivers’ CES-D score was slightly higher at three-month post-stroke (4, IQR 2–7), as compared to one-year post-stroke (3, IQR 1–5), by one point. The median caregivers’ ZBI score was also higher at three-month post-stroke (7, IQR 5–13) than one-year post-stroke (5, IQR 0–11) by two points. For the quality of care-relationship, the medians were the same at three-month (12, IQR 10–12) and one-year post-stroke (12, IQR 9–12). In the case of expressive social support, the difference in the median was one point, which was higher at three-month (25: IQR 23.5–32) than one-year post-stroke (24, IQR 23–29). The continuous variables in Table 2 had high internal consistency, as the values of Cronbach’s alpha were between 0.76 and 0.95.

**Table 2:** Summary statistics of CES-D score and time-varying variables

		Three-month (*n* = 214)^a^	Cronbach’s alpha for three-month	One-year (*n* = 143)^b^	Cronbach’s alpha for 1-year
**Caregivers**					
Median CES-D (IQR)		4 (2–7)	0.84	3 (1–5)	0.76
Median ZBI Score (IQR)		7 (5–13)	0.82	5 (0–11)	0.88
Median quality of care-relationship (IQR)		12 (10–12)	0.89	12 (9–12)	0.95
Median expressive social support (IQR)		25 (23.5–32)	0.95	24 (23–29)	0.93
Median OCBS (IQR)		31 (23–42)	0.92	29 (19–37)	0.95
Number of health conditions, n (%)					
	No health conditions	151 (72.3%)		125 (87.4%)	
	1 or more health condition	58 (27.8%)		18 (12.6%)	
**Patients**					
Median CES-D (IQR)		4.5 (1–9)	0.88	4 (1–7)	0.80

CES-D (Center for epidemiologic studies depression), *n* (sample size), IQR (Interquartile range), OCBS (Oberst caregiving burden score), ZBI (Zarit Burden Interview)

^a^Number of missing observations: Caregivers’ CES-D (n = 5), ZBI (n = 3), Expressive Social Support (n = 2), OCBS (n = 35), number of health conditions (n = 5), Patients’ CES-D (n = 14)

^b^Number of missing observations: Caregivers’ CES-D (n = 2), ZBI (n = 3), Expressive Social Support (n = 2), OCBS (n = 6), Patients’ CES-D (n = 2)

### Variables associated with caregivers’ depressive symptoms

Table 3 shows the unadjusted and adjusted Tobit regression models for caregivers’ depressive symptoms. For unadjusted bivariate analysis, the following variables related to caregivers were significant: subjective burden, quality of care-relationship, expressive social support, number of health conditions and ethnicity (Malay vs Chinese). Significant patient-related variables included ethnicity (Malay vs Chinese) and CES-D score.

**Table 3:** Mixed-effect Tobit regression (outcome: CES-D of caregiver)

		Unadjusted model		Adjusted model	
		Partial regression coefficient (95% CI)	*P* value	Partial regression coefficient (95% CI)	*P* value
**Variable related to caregivers**					
Subjective burden (ZBI)		0.27 (0.21 to 0.33)	** < 0.001**	0.18 (0.11 to 0.24)	** < 0.001**
Quality of care-relationship		− 0.61 (− 0.91 to − 0.31)	** < 0.001**	− 0.35 (− 0.63 to − 0.06)	**0.017**
Expressive social support		− 0.39 (− 0.50 to − 0.29)	** < 0.001**	− 0.28 (− 0.37 to − 0.19)	** < 0.001**
Objective burden (OCBS)		0.07 (0.04 to 0.11)	** < 0.001**	0.00 (− 0.03 to 0.03)	0.930
Number of health conditions	No health condition (reference)				
	1 or more health condition	1.65 (0.47 to 2.84)	**0.006**	0.79 (− 0.24 to 1.82)	0.132
Age		0.02 (− 0.02 to 0.07)	0.326		
Gender	Male (reference)				
	Female	0.90 (− 0.39 to 2.19)	0.172	0.36 (− 0.79 to 1.50)	0.542
Ethnicity	Chinese (reference)				
	Malay	− 1.72 (− 2.96 to − 0.47)	**0.007**	0.23 (− 0.76 to 1.22)	0.652
	Indian	0.49 (− 1.58 to 2.55)	0.644	1.83 (0.21 to 3.44)	**0.026**
	Others	1.93 (− 1.60 to 5.45)	0.285	3.00 (0.60 to 5.40)	**0.014**
Educational qualification	No qualification (Reference)				
	Primary	− 1.62 (− 4.62 to 1.39)	0.292		
	Secondary	− 1.70 (− 4.60 to 1.19)	0.249		
	Post-secondary/polytechnic	− 3.00 (− 6.01 to 0.01)	0.050		
	University	− 2.57 (− 6.14 to 1.00)	0.158		
Employment status	Employed full time (reference)				
	Employed part time	1.31 (− 0.73 to 3.35)	0.207		
	Unemployed	0.73 (− 0.50 to 1.95)	0.245		
Relationship with patient	Spousal (reference)				
	Children	− 0.37 (− 1.62 to 0.87)	0.555	0.76 (− 0.34 to 1.86)	0.174
	Others	− 0.04 (− 2.04 to 1.95)	0.966	− 0.74 (− 2.40 to 0.92)	0.381
Time period	3rd month (reference)				
	1st year	− 1.44 (− 2.28 to − 0.60)	**0.001**	− 1.00 (− 1.80 to − 0.20)	**0.014**
**Variable related to patient**					
Age		0.03 (− 0.02 to 0.09)	0.183	0.02 (− 0.03 to 0.06)	0.422
Gender	Male (reference)				
	Female	− 0.73 (− 1.93 to 0.46)	0.228	− 0.64 (− 1.78 to 0.51)	0.275
Ethnicity^a^	Chinese (reference)				
	Malay	− 1.49 (− 2.75 to − 0.22)	**0.021**		
	Indian	0.05 (− 1.92 to 2.02)	0.959		
	Others	2.00 (− 1.83 to 5.83)	0.307		
CES-D		0.33 (0.25 to 0.40)	** < 0.001**	0.16 (0.09 to 0.23)	** < 0.001**

*p* < 0.05 are indicated in bold

CES-D, Center for Epidemiologic Studies Depression; CI, confidence interval; OCBS, Oberst Caregiving Burden Score; ZBI, Zarit burden interview

^a^Overall was not significant, hence was not included in the adjusted model

The adjusted model showed that all variables of interest were significantly associated with caregivers’ depressive symptoms. For every one-point increase in ZBI score, the caregivers’ CES-D score increased by 0.18 (95% 0.11–0.24) points. When the quality of care-relationship increased by one point, the caregivers’ CES-D score decreased by 0.35 (95% CI − 0.63 to − 0.06) points. The caregivers’ CES-D score reduced by 0.28 (95% CI − 0.37 to − 0.19) points for every one-point increase in expressive social support.

Besides the variables of interest, the following control variables were significant in the multivariable model: caregivers’ ethnicity (Indian/Others vs Chinese) and patients’ CES-D score. The effect of time was also significant in the adjusted model, with one-year post-stroke having a lower CES-D score than three-month post-stroke (partial regression coefficient: − 1.00, 95% CI − 1.80 to − 0.20). OCBS was not significantly associated with caregivers’ CES-D score in the adjusted model.

## Discussion

By knowing the determinants of caregivers’ depressive symptoms, healthcare providers can teach caregivers relevant skills to cope with their depressive symptoms. Our results showed that caregivers’ depressive symptoms were similar between three-month and one-year post-stroke before adjustment. However, after controlling for confounders, caregivers' depressive symptoms were higher at three months post-stroke than one year later. Investigating the associations with variables of interest showed that subjective (but not objective) burden correlated positively with caregivers' depression symptoms. Moreover, good patient-caregiver relationships and good expressive social support were significantly associated with lower caregivers' depressive symptoms.

Several quantitative studies corroborated our findings that the trend of caregivers' depressive symptoms did not differ over time without adjustment. In a study at Helsinki University Central Hospital, the prevalence of caregivers’ depressive symptoms decreased from 33% at the acute phase to 30% at 6-month post-stroke [4]. However, the prevalence remained the same in six months and 18 months following stroke [4]. A study in Singapore by Malhotra et al. [8] also presented minute changes in caregivers’ depressive symptoms over time. Based on a 20-item CES-D score, the study found that the score increased by 2.1 points from 0–10 weeks to 11–22 weeks following stroke [8]. There was also a slight decrease by 2.1 points from 11–22 weeks to 23–47 weeks after stroke [8].

Nonetheless, our findings showed a significant decrease in caregivers' depressive symptoms from three-month to one-year post-stroke after adjustment. Although there is a lack of quantitative studies to compare this finding, several qualitative studies on stroke may empirically explain the similar findings from our study. According to a qualitative study on caregivers’ experience with incontinence in stroke survivors, this phenomenon may be attributed to coping strategies adopted by caregivers over time [36]. Caregivers become more confident in their role by learning how to handle incontinence and adjusting their perception of excretion [36]. Another qualitative study also found that in the process of readjustment, caregivers were able to gain network support and navigate their roles better with skills such as multitasking [36]. Hence, adopting appropriate coping strategies may help caregivers adjust to their responsibilities and promote their psychological well-being over time.

Our study found a positive association between subjective burden and caregivers’ depressive symptoms. While these two concepts may seem similar, they have distinct definitions. Subjective burden refers to how caregivers internally perceive the consequences of caregiving [37]. Caregivers’ depressive symptoms is defined as a mood disturbance caused by the pressure of caregiving [38]. In the systematic review by del-Pino-Casado et al. [14], the pooled effect size of five stroke-related studies similarly showed a significant positive correlation (r = 0.416, 95% CI 0.331–0.494). According to another stroke-related quantitative study, the odds of caregiver’s depression was 2.9 times higher for a unit increase in caregiver’s burden (measured using caregiver’s strain index) after adjustment [30]. Despite these similar findings in the literature, there was no existing explanation for this association. Future studies need to examine the dynamic mechanisms between subjective burden and caregivers’ depressive symptoms.

Our result also revealed that good patient-caregiver relationships were negatively associated with caregivers’ depressive symptoms. This finding corroborates a quantitative study by Kruithof et al. [13] on partners’ burden, anxiety and depressive symptoms among partners of stroke survivors. According to the study, partners who were satisfied with their relationship two months post-stroke were less likely to be depressive [13]. Our finding may be explained from an Asian socio-cultural perspective. A qualitative study from China found that caregivers viewed their role as an obligation and expressive of love [39]. Another qualitative study on Chinese caregivers unveiled that caregiver was able to spend more time with the patients following a stroke event [40]. Moreover, caregivers became more reliable and sensible in the process [40]. These benefits acquired from having good patient-caregiver relationships may negate caregivers’ depressive symptoms.

Another variable negatively associated with caregivers’ depressive symptoms was expressive social support. Although there is a lack of quantitative studies on the association between caregivers’ depressive symptoms and expressive social support in stroke settings, similar findings were found in quantitative studies from other settings [26]. In a Singapore-based study on caregivers of older adults, caregivers with expressive social support were less likely to have depressive symptoms [26]. This association is possible because perceived social support may provide a sense of social security for the caregivers [13]. A qualitative study mentioned that caregivers might suffer negative emotions due to fatigue from responsibilities and feeling out of control [41]. In such situations, social support may be helpful to ensure that they feel secure and connected with the community [41, 42].

By identifying these protective factors of caregivers’ depressive symptoms, healthcare providers can tailor more effective psychosocial interventions to improve caregivers’ outcomes. So far, researches on psychosocial interventions have mainly focused on problem-solving and stress-coping strategies [43]. These methods were shown to enhance caregivers’ psychological well-being and reduce the utilisation of healthcare resources [43]. Our findings suggests that teaching communication skills to caregivers may be pertinent as well. It will improve caregivers’ relationships with patients and allow caregivers to leverage social support. Some caregivers also may not be aware of the benefits of perceived social support [44]. An Asian study had alluded that caregivers might avoid seeking support from their networks [40]. In these situations, healthcare providers may explain the benefits of perceived social support to the caregivers.

Although objective burden was not a variable of interest, it was surprising to find no significant association between objective burden and caregivers’ depressive symptoms after adjustment. Moreover, the result showed that the association may be too small to be considered relevant (partial regression coefficient: 0.00, 95% CI − 0.03 to 0.03). There are two possible reasons for this finding. Firstly, since the study sample did not include patients with severe stroke, the caregiving demand may be relatively manageable. Hence, it is likely that this association may only be observed for caregivers taking care of patients with severe conditions. Secondly, caregivers’ depressive symptoms may be associated with the difficulty of the tasks instead of the time spent. In a study by Grant et al. [6], the odds of caregivers’ depressive behaviour increased by 5% for a unit increase in objective burden. This study utilised the difficulty sub-scale of the Caregiving Burden scale [6] but our study only measured the time aspect of OCBS.

Our study has several strengths. It contributes to the number of studies that have examined the association between caregivers’ psychosocial characteristics and caregivers’ depressive symptoms in an Asian stroke setting. Identifying protective factors of caregivers’ depressive symptoms also revealed that caregivers’’ communication skills might be salient in improving their outcomes. Our study used a longitudinal dataset, which allowed the inclusion of time-varying covariates in the regression model. Moreover, we attempted to adjust for established confounders in the regression model.

However, our study is not without limitations. Our study model was established from the existing data, limiting the addition of other relevant confounders, such as caregivers’ self-efficacy [13], which were not collected and unadjusted for in the model specification. In addition, it may be difficult to generalise our findings beyond the Asian setting, as the sample was from Singapore. There may also be selection bias. Compared with caregivers included in the analysis, caregivers excluded were younger, of Chinese ethnicity, held university degrees, worked part-time and were neither spouses nor children of stroke survivors. Moreover, 71 caregivers were excluded at one-year post-stroke due to a change in caregiver status or lost-to-follow-up. This exclusion may reduce the power of the study and the sample’s representativeness of the population. The Pearlin's Stress Process Model, as previously mentioned, has guided us to explain the theoretical relationships between caregivers' psychosocial characteristics and depressive symptoms found in this study. However, our research design was limited in establishing causality because we collected the independent variables and outcome variables concurrently.

## Conclusion

Our study investigated the association between caregivers’ depressive symptoms and caregivers’ psychosocial characteristics among caregivers of patients with stroke. We found that subjective burden had a positive association with caregivers’ depressive symptoms. Moreover, having good caregiver-patient relationships and more expressive social support had negative associations with caregivers’ depressive symptoms. As a result, for caregivers of patients with stroke, more specialized interventions for family-focused communication skills and stroke caregiver support groups, as well as family counselling for caregivers, may improve caregivers' relationships with patients and enable caregivers to tap into social support. Healthcare providers can also explain the benefits of expressive social support to these caregivers who do not know the benefits.

## Supplementary Information

**Additional file 1: Fig. S1.** CES-D of caregivers for (a) three-month post-stroke and (b) one-year post-stroke.

## Data Availability

The datasets used during the current study are available from the corresponding author on reasonable request. The questionnaires utilized in the study can be provided by contacting the corresponding author.
