# Supplementary material for: The associations between caregivers’ psychosocial characteristics and caregivers’ depressive symptoms in stroke settings: a cohort study
Source: BMC Psychol. 2022 May 9;10:121. doi: 10.1186/s40359-022-00828-2 (PMC9082830; doi:10.1186/s40359-022-00828-2)

**Supplementary Figure 1**: CES-D of caregivers for (a) three-month post-stroke and (b) one-year post-stroke


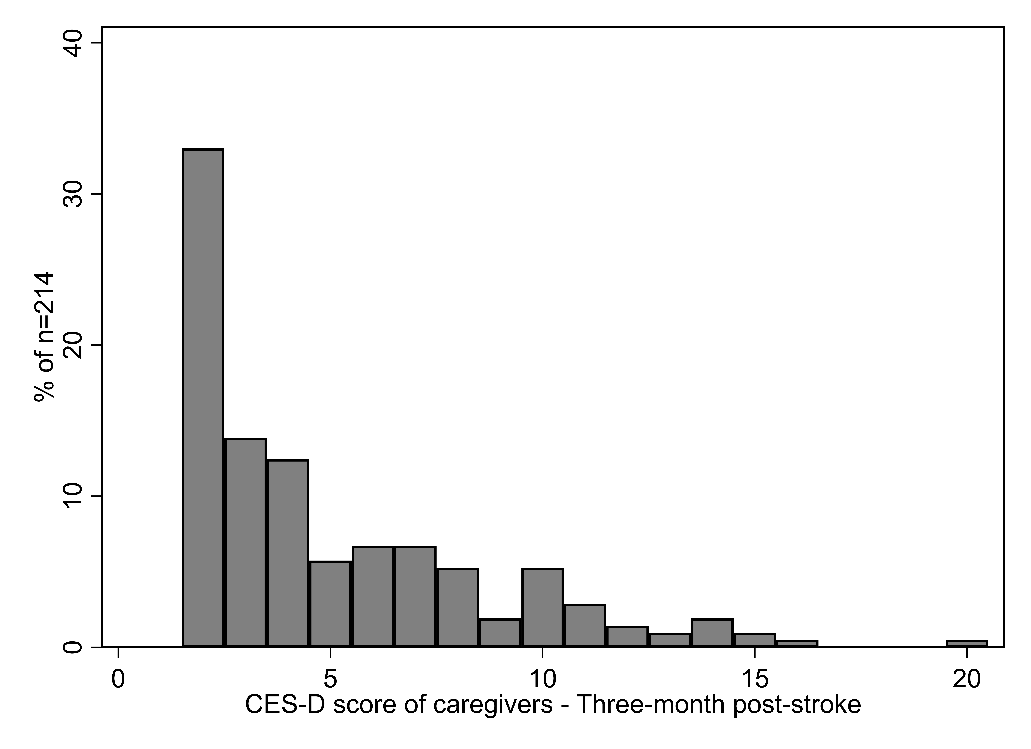


(b)


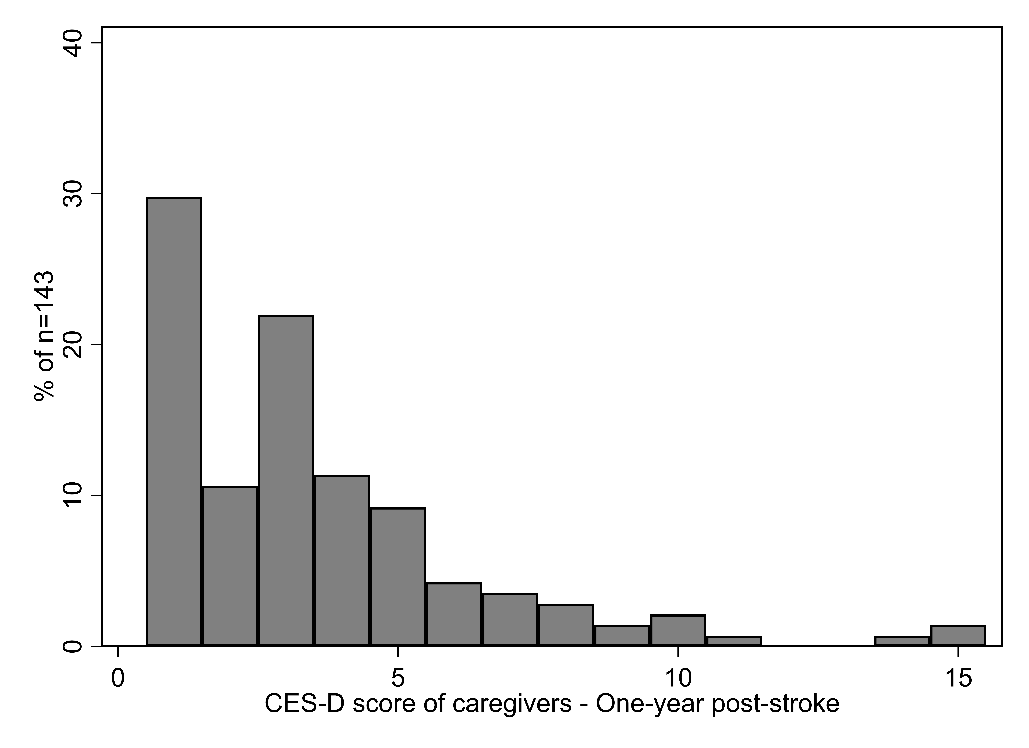

Supplement: Supplementary file 1 — Additional file 1: Fig. S1. CES-D of caregivers for (a) three-month post-stroke and (b) one-year post-stroke. [file 40359_2022_828_MOESM1_ESM.docx]
